# Retraction Note: Undertreatment of breast cancer in the elderly

**DOI:** 10.1186/s12893-016-0140-7

**Published:** 2016-04-28

**Authors:** Nicola Rocco, Corrado Rispoli, Gennaro Pagano, Silvio Ascione, Rita Compagna, Michele Danzi, Antonello Accurso, Bruno Amato

**Affiliations:** Department of Biomedical, Surgical and Dental Sciences, University of Milan, Milan, Italy; Department of General Surgery, Cardinale Ascalesi Hospital - ASL NA1, Naples, Italy; Department of Translational Medical Sciences, University “Federico II” of Naples, Naples, Italy; Private Practice, Palazzo Donn’Anna, Largo Donn’Anna 9, Naples, Italy; Department of General, Geriatric, Oncologic Surgery and Advanced Technologies, University “Federico II” of Naples, Naples, Italy

## Retraction Notice

This article [1] has been retracted by the authors due to significant overlap with a previous publication by Malik et al. [2]. Gennaro Pagano was not involved in the study and was introduced into the author list in error by the corresponding author. All authors, including Gennaro Pagano, have subsequently agreed that he did not qualify for authorship. The remaining authors apologize for any inconvenience caused.

## Notes

The online version of the original article can be found at 10.1186/1471-2482-13-S2-S26.

## References

[1] Rocco N, Rispoli C, Pagano G, Ascione S, Compagna R, Danzi M, Accurso A, Amato B (2013). Undertreatment of breast cancer in the elderly. BMC Surg.

[2] Malik MK, Tartter PI, Belfer R (2013). Undertreated breast cancer in the elderly. J Cancer Epidemiol.

